# microRNAs: Emerging Targets Regulating Oxidative Stress in the Models of Parkinson's Disease

**DOI:** 10.3389/fnins.2016.00298

**Published:** 2016-06-28

**Authors:** Yangmei Xie, Yinghui Chen

**Affiliations:** ^1^Department of Neurology, Jinshan Hospital, Fudan UniversityShanghai, China; ^2^Department of Neurology, Shanghai Medical College, Fudan UniversityShanghai, China

**Keywords:** oxidative stress, microRNAs, Parkinson's disease, mitochondrial dysfunction, α-synuclein, Nrf2

## Abstract

Parkinson's disease (PD) is the second most common neurodegenerative disorder. This chronic, progressive disease is characterized by loss of dopaminergic (DA) neurons in the substantia nigra pars compacta (SNpc) and the presence of cytoplasmic inclusions called Lewy bodies (LBs) in surviving neurons. PD is attributed to a combination of environment and genetic factors, but the precise underlying molecular mechanisms remain elusive. Oxidative stress is generally recognized as one of the main causes of PD, and excessive reactive oxygen species (ROS) can lead to DA neuron vulnerability and eventual death. Several studies have demonstrated that small non-coding RNAs termed microRNAs (miRNAs) can regulate oxidative stress *in vitro* and *in vivo* models of PD. Relevant miRNAs involved in oxidative stress can prevent ROS-mediated damage to DA neurons, suggesting that specific miRNAs may be putative targets for novel therapeutic targets in PD.

## Introduction

**Figure 1:**
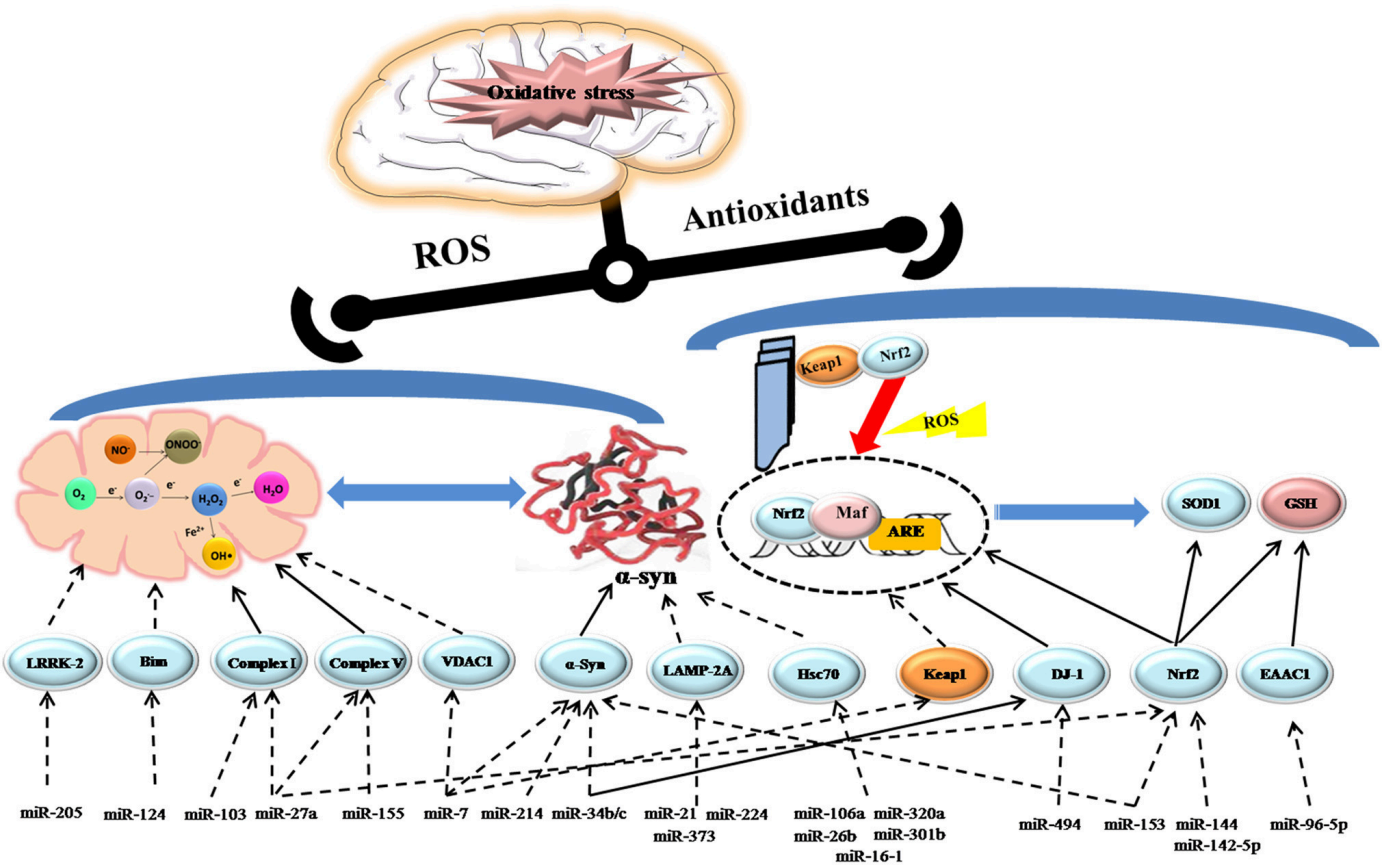
**Schematic model depicting the role of miRNAs regulating oxidative stress in PD**. Activation steps are represented by solid lines and inhibitory effects are represented by dashed lines.

PD is the most common degenerative movement disorder affecting 1–2% of individuals older than 65 (de Lau and Breteler, 2006). It is clinically characterized by motor symptoms including resting tremor, muscle rigidity, bradykinesia, and general postural instability. Obviously, the patients of PD gradually suffer from severe motor and cognitive disability in the years after diagnosis, which imposes a considerable burden on both families and society (Savitt et al., 2006). In PD, the imbalance between ROS production and the antioxidant system results in oxidative stress. Superoxide (O^2−^), hydrogen peroxide (H_2_O_2_), and the hydroxyl radical (OH∙), the main biologically ROS in mammalian cells can damage macromolecules including nucleic acids, lipids and proteins, leading to dopaminergic (DA) neuron degeneration, and neuron network dysfunction, ultimately progressing to PD (Sanders and Greenamyre, 2013). It is well accepted that oxidative stress contributes to PD, and there is interest in understanding how oxidative stress is exacerbated by the emerging regulators, microRNAs (miRNAs) via contributing to the pathological processes which are responsible for oxidative stress like mitochondrial dysfunction (Subramaniam and Chesselet, 2013), α-synuclein aggregation (Hsu et al., 2000), neuroinflammation (Tansey et al., 2007), and dysregulation of the endogenous antioxidant system (Buendia et al., 2016). microRNAs are highly conserved noncoding RNAs, ~18–25 nucleotides in length that can regulate protein expression either by translational inhibition or targeted mRNA cleavage (Bartel, 2009; Guo et al., 2010). miRNA transcripts are produced in the nucleus and then processed into hairpin RNA (pre-RNA) by the Drosha microprocessor complex. Exprotin-5 transfers pre-RNA out of the nucleus for final processing to mature RNA by the RNAase III enzyme Dicer. Mature RNA forms the RNA-induced silencing complex (RISC) with Argonaute (Ago) proteins, which can form sequence-specific base-pairing with target mRNA, usually with the 3′-untranslated region (UTR). The formation can regulate target gene expression through mRNA degradation or translational inhibition, in which polymorphisms in the 3′- UTR of target mRNA may play a role (Ghanbari et al., 2016). Actually, one miRNA may have multiple mRNA targets, so that it may involve in diverse pathological processes. Meanwhile, the 3′-UTR of target mRNA can be recognized by multiple miRNAs and they can alter cellular responses via a coordinated network (Filipowicz et al., 2008). It has recently been shown that miRNAs are involved in different pathways to regulate cellular redox responses and participate in the pathophysiological processes of PD (seen in Figure 1). In this review, we will highlight the significant roles of miRNAs in oxidative stress contributing to pathogenesis of PD in models of PD.

## miRNAs and mitochondrial dysfunction

Oxidative phosphorylation is the main mechanism by which energy (ATP) is produced to power neural activity (Sterky and Larsson, 2008). However, when the normal function of mitochondria is impaired, leakage of electrons from the respiratory chain may directly promote ROS production, eventually exacerbating neuronal dysfunction. Mitochondrial dysfunction and reduced complex I activity are observed in the SNpc and frontal cortex of PD patients (Parker et al., 2008). Several studies have implied that miRNAs may serve as potential biomarkers and provide a possible therapeutic avenue to alleviate mitochondrial dysfunction in PD. It has been reported that miR-124 is downregulated in DA neurons of MPTP (1-methyl-4-phenyl-1,2,3,6-tetrahydropyridine)-induced mice and in MPP+(1-methyl-4-phenylpyridinium)-treated SH-SY5Y cells, respectively. Conversely, miR-124 upregulation can inhibit expression of the protein Bim, and reduce translocation of its downstream protein Bax to mitochondria and lysosome, thus suppressing mitochondria apoptotic signaling pathways and normalizing the impaired autophagy process (Wang et al., 2016). Gene mutations also can be blamed for mitochondrial dysfunction (Ryan et al., 2015). Mutations in PARK7, the encoding gene of protein DJ-1can cause autosomal recessive PD characterized by decreased DJ-1 levels in the SNpc. Numerous studies have demonstrated that DJ-1 can directly bind to the subunits of complex I and maintain its activity as an integral mitochondrial protein (Hayashi et al., 2009). Mitochondrial morphology and dynamics are clearly damaged in DJ-1-knockdown neurons (Irrcher et al., 2010). A recent study indicated that miR-494 could increase oxidative stress-induced neuronal lesions by inhibiting DJ-1 expression (Xiong et al., 2014). Another study has proposed that reduced miR-34b/c levels along with decreased expression of DJ-1 could contribute to abnormal mitochondrial defects in the brain of PD patients, which is confirmed in miR-34b/c-depleted cells. However, miR-34b/c may modulate DJ-1 expression in an indirect way and the specific mechanism needs to be further clarified (Minones-Moyano et al., 2011). Mutation in leucine-rich repeat kinase 2 (LRRK2) is another identifiable cause for autosomal dominant PD. Increased LRRK2 protein levels can compromise mitochondrial dynamics and integrity through a fission protein, called dynamin-like protein (DLP1; Mortiboys et al., 2010; Wang et al., 2012). Interestingly, miR-205 expression is significantly downregulated in the brains of PD patients, accompanied by elevated LRRK2 protein levels. Further studies revealed that miR-205 could suppress LRRK2 protein expression in primary neuron cultures by targeting the 3′-UTR of LRRK2 gene (Cho et al., 2013). Moreover, miRNAs exhibit a critical role in inflammation-mediated mitochondrial dysfunction in PD. Following treatment of SH-SY5Y cells with tumor necrosis factor-α, increased miR-27a and miR-103 could suppress expression of the functional units of complexI and higher levels of miR-155 and miR-27a could result in mitochondrial defects and oxidative stress via downregulating transcript levels of ATP5G3, a subunit of complexV (Prajapati et al., 2015). In addition, miR-7 has been found to stabilize mitochondrial membrane potential via suppressing the expression of voltage-dependent anion channel 1 (VDAC1), one constituent of the mitochondrial permeability transition pore, which might be a potential target for attenuating the impaired mitochondrial function in PD (Chaudhuri et al., 2016).

## miRNAs and α-synuclein aggregation

α-synuclein (α-Syn) is the main component of the LBs, and its presence in these structures correlates with non-motor manifestations of PD, including autonomic, sleep, and olfactory dysfunction. Point mutations and multiplications in the SNCA gene locus in familial PD, are directly associated with high levels of α-Syn mRNA and proteins in the frontal cortex (Venda et al., 2010). It is generally accepted that oxidative stress can trigger the expression of α-Syn and increase its oligomerization and fibrillization, subsequently aggravating its neurotoxicity. In the contrast, aggregated α-syn species can impair mitochondrial structure and inhibit complex I activity, thus promoting ROS production (Hsu et al., 2000). In the course of PD, the two processes form a vicious circle and accelerate progression of the disease. Currently, it has been suggested the decreased level of miR-7 and miR-214 possibly contributes to increased α-Syn accumulation in the MPTP-induced model of PD (Junn et al., 2009; Wang et al., 2015). Another study has further confirmed that mir-7 and mir-153 could synergistically downregulate α-Syn expression post-transcriptionally (Doxakis, 2010). Additionally, one study has revealed that the decreased level of miR-34b/c in specific brain areas of PD patients, including the amygdala, frontal cortex, substantia nigra, and cerebellum, might contribute to PD pathogenesis for losing its function of inhibiting α-Syn expression (Kabaria et al., 2015b). miRNAs not only can regulate α-Syn synthesis, but can also impair its degradation pathway known as chaperone-mediated autophagy (CMA). CMA pathway for removing exceptional α-Syn mainly includes two proteins: lysosomal-associated membrane protein 2A (LAMP-2A) and heat shock protein 70 (hsc70). It has been suggested that increased miR-21, miR-224, and miR-373 in the SNpc could inhibit LAMP-2A expression, while enhanced levels of miR-26b, miR-106a, and miR-301b could suppress hsc70 expression, resulting in α-Syn deposition by inhibiting its degradation (Alvarez-Erviti et al., 2013). Furthermore, other studies have demonstrated that miR-320a and miR-16-1 could promote aberrant α-Syn accumulation by targeting hsc70 in PD (Li et al., 2014; Zhang and Cheng, 2014).

## miRNA and the endogenous antioxidant system

As an endogenous mechanism against oxidative stress, the nuclear factor erythroid 2-related factor 2-antioxidant-response element (Nrf2-ARE) pathway is deregulated as PD progresses (Ramsey et al., 2007). It is primarily regulated by its inhibitory protein -Kelch-like ECH-associated protein 1 (Keap1), which sequesters Nrf2 in the cytoplasm and facilitates its proteasomal degradation, maintaining a low level of Nrf2 in cytoplasm. Under oxidative stress, the transcription factor may translocate to the nucleus and activate the expression of its downstream ARE such as NADPH quinine oxidoreductase 1 (NQO-1), heme oxygenase-1 (HO-1), superoxide dismutase (SOD), and glutathione (GSH), etc (Satoh et al., 2006; Zhang et al., 2013). Treated with the agonist of the Nrf2-ARE pathway, the nigrostriatal neurons exhibit greater resistance to MPTP-induced neurotoxicity (Yang et al., 2009). Brain-enriched miR-7 can repress Keap1 expression, which leads to increased Nrf2 activity and upregulation of HO-1 expression in SH-SY5Y cells (Kabaria et al., 2015a). Moreover, it was found that DJ-1, the stabilizer of Nrf2 could upregulate the level of SOD1 through activating the Erk1/2–Elk1 pathway (Wang et al., 2011). Meanwhile, DJ-1 is the direct target of miR-494 as aforementioned (Xiong et al., 2014), so decreasing the level of miR-494 might protect neurons against oxidative insult by promoting SOD1expression as well as attenuating mitochondrial impairment. Furthermore, the Nrf2-ARE pathway is directly regulated by various protein kinases like protein kinase C (PKC) and glycogen synthase kinase 3 beta (GSK-3b) or epigenetic factors like miRNAs and promoter methylation, which are independent of Keap1 (Bryan et al., 2013). Recently, it was reported that miR-153, miR-27a, miR-142-5p, and miR-144 could directly downregulate Nrf2 expression in SH-SY5Y neuronal cells, thereby deregulating the ARE/GSH pathway and disturbing GSH homeostasis in the brain (Narasimhan et al., 2012). GSH is the most indispensable free radical scavenger in the central nervous system, and a decreased GSH/GSSG ratio is considered as a pathogenic factor in PD. Rhythmic expression of the cysteine transporter excitatory amino acid carrier 1 (EAAC1), an important regulator of GSH synthesis, is negatively regulated by miR-96-5p, which also displays a diurnal rhythm. In addition, blocking miR-96-5p with an inhibitor can increase the levels of EAAC1 and GSH, exerting a neuroprotective effect against ROS in the mouse SN (Kinoshita et al., 2014).

## Conclusion

Although numerous studies have provided evidence that miRNAs play a role in PD pathogenesis by regulating oxidative stress, the field is still in its infancy. Actually, the causal factors inducing oxidative stress in PD connect and interact with each other rather than function independently, which make it difficult to elucidate the function of related miRNAs totally. In addition, accumulating evidences mainly confirmed from animal and cell models, and related miRNA studies in PD patients are limited to the difference expression in the brain (Table 1). To date, it is not clear whether specific miRNAs can directly induce PD. As the field matures, understanding how miRNAs can mediate oxidative stress in PD could lead to the development of new PD therapies.

**Table 1:** **microRNAs profiling involved in oxidative stress in PD**.

**Human studies/Experiment models**	**Upregulated miRNAs**	**Downregulated miRNAs**	**Rhythmic miRNAs**
Experiment models	miR-494 miR-153 miR-142-5p	miR-124 miR-214 miR-7	miR-96-5p
	miR-27a miR-103 miR-155	miR-153	
PD patients	miR-320a miR-26b miR-301b	**miR-34b/c** **miR-205**	
	miR-21 miR-224 miR-373		

*miR-34b/c, miR-205 were both decreased in PD patients and experiment models, which are highlighted*.

## Author contributions

YX and YC conceived the article and wrote the manuscript. YC reviewed and edited the manuscript. All authors read and approved the manuscript.

### Conflict of interest statement

The authors declare that the research was conducted in the absence of any commercial or financial relationships that could be construed as a potential conflict of interest.
